# Sero-epidemiological profiling of duck viral hepatitis, avian influenza, and Newcastle disease in transboundary waterfowl populations along the Indonesia–Malaysia border of West Kalimantan

**DOI:** 10.14202/vetworld.2026.2160-2171

**Published:** 2026-05-21

**Authors:** Eny Martindah, Sutiastuti Wahyuwardani, Sri Suryatmiati Prihandani, Harimurti Nuradji, Difa Widyasari, Ahmad Mike Ariyanto, Raphaella Widiastuti, Rini Damayanti, Atik Ratnawati, Andriani Andriani, Muharam Saepulloh

**Affiliations:** 1 Research Center for Veterinary Science, National Research and Innovation Agency, Cibinong Science Center, Cibinong, Indonesia; 2 Center for Diagnostic Standards of Agricultural Quarantine Laboratory Test Service, Jakarta Timur, Indonesia; 3 Plantation and Livestock Agency of West Kalimantan Province, Pontianak, Indonesia

**Keywords:** avian influenza, duck viral hepatitis, Newcastle disease, One Health, sero-epidemiology, transboundary surveillance, waterfowl, West Kalimantan

## Abstract

**Background and Aim:**

Waterfowl, particularly *Anas platyrhynchos* and *Cairina moschata*, are important reservoirs and amplifiers of transboundary viral diseases, including Duck viral hepatitis (DVH), Avian influenza (AI), and Newcastle disease (ND). Despite their epidemiological significance, data from the Indonesia–Malaysia border region of West Kalimantan remain scarce. This study aimed to determine the seroprevalence and epidemiological patterns of DVH, AI, and ND in waterfowl populations in this high-risk transboundary interface.

**Materials and Methods:**

A cross-sectional study was conducted in Sambas, Bengkayang, and Sanggau districts using purposive sampling. A total of 211 serum samples from ducks and Muscovy ducks across 26 backyard flocks were analyzed. Antibodies against DVH were detected using an enzyme-linked immunosorbent assay, whereas AI and ND antibodies were assessed using hemagglutination inhibition tests following World Organization for Animal Health guidelines. Clinical examination, gross pathology, and histopathology were performed to support serological findings. Statistical analyses included prevalence estimation, Chi-square tests, and univariate logistic regression, with significance set at p < 0.05.

**Results:**

No serological evidence of DVH was detected across all districts, species, or age groups, indicating an apparent absence of exposure in the study population. In contrast, AI and ND antibodies were detected with overall seroprevalence of 5.69% and 13.27%, respectively. ND showed a relatively uniform distribution across districts, whereas AI seroprevalence differed significantly, with the highest detection in Sanggau. Age-related trends suggested increased ND exposure in older birds, whereas AI exposure was higher in younger birds; however, these differences were not statistically significant. Clinicopathological findings, including hemorrhagic lesions and necrosis, were consistent with viral infections, particularly ND and AI, and supported serological evidence of circulating pathogens.

**Conclusion:**

The absence of DVH and the presence of AI and ND antibodies indicate differential circulation dynamics of major viral pathogens in waterfowl along the Indonesia–Malaysia border. These findings highlight the need for strengthened cross-border surveillance, improved vaccination strategies, and integrated One Health approaches to mitigate transboundary disease risks in backyard production systems.

## INTRODUCTION

Waterfowl, notably ducks (*Anas platyrhynchos*) and Muscovy ducks (*Cairina moschata*), play a crucial role in local economies and ecosystems, particularly in regions such as West Kalimantan. However, these birds are vulnerable to several viral diseases that can cause significant economic and ecological damage. Viral infections have substantial impacts on duck production systems [1]. Among the most important viral diseases are duck viral hepatitis (DVH), avian influenza (AI), and Newcastle disease (ND), all of which are associated with high mortality and rapid transmission in waterfowl populations. Duck viral hepatitis (DVH), caused by Duck hepatitis A virus (DHAV), is especially alarming due to its rapid onset and high mortality rates in affected flocks [2]. Ducklings are most susceptible to DHAV infection when they are less than four weeks old, and their resistance increases gradually with age [1, 3]. The disease typically begins suddenly, spreads rapidly through the flock, and may cause mortality rates of up to 95% [4, 5]. Despite its severity, there are limited reports of DVH in Indonesia, although cases have been documented in Southeast Asia. Evidence indicates that DHAV-1 outbreaks occur globally, whereas DHAV-2 and DHAV-3 are primarily reported in East and South Asia [6, 7]. Epidemiological studies from China have demonstrated that DHAV-1 remains the predominant genotype. The genetic evolution of DHAV strains is closely associated with geographic distribution, reflecting regional viral diversity [8]. Consequently, the vaccine strains used for DHAV may not always correspond to circulating serotypes, potentially leading to inadequate immune protection [9].

These concerns are particularly relevant in border regions such as West Kalimantan, which shares a porous boundary with the Malaysian state of Sarawak. The prevalence of ND and AI in these areas further complicates the epidemiological situation. AI is well recognized for its zoonotic potential, and together these diseases impose severe economic losses on poultry production systems. Assessing the sero-epidemiology of these diseases in border regions is critical for developing effective preventive measures and early detection strategies. The transmission dynamics of H5N1 between domestic ducks and migratory birds in agroecological environments are influenced by several interrelated factors. Agroecological conditions and the presence of migratory birds contribute to the emergence and persistence of H5N1 in Southeast Asia [10]. Stopover duration during migration and interactions at the wild–domestic interface further shape transmission dynamics [11]. Hill *et al*. [12] reported that the overlap between wild and domestic birds, particularly during migration, facilitates viral reassortment and increases the potential for new outbreaks, whereas Damodaran *et al*. [13] demonstrated that backyard flocks often serve as early indicators of H5N1 presence due to their proximity to wild birds.

Investigating the sero-epidemiology of infectious diseases in waterfowl, including DVH, AI, and ND, is essential for understanding disease dynamics and mitigating risks to poultry production and public health in border regions. Although ND and AI exposure have been documented in several parts of Indonesia, particularly in Java and other central regions [14, 15], surveillance data from transboundary areas remain limited. Furthermore, reports of DHAV circulation in Indonesia are scarce, with most published data originating from East and Southeast Asia.

Despite the recognized importance of DVH, AI, and ND in waterfowl health and their substantial economic and public health implications, there is a critical lack of integrated epidemiological data from transboundary regions such as West Kalimantan. Existing studies in Indonesia have predominantly focused on single pathogens or geographically isolated regions, with limited emphasis on multi-pathogen surveillance in border ecosystems where cross-border animal movement, shared ecological niches, and interactions between domestic and wild birds significantly influence disease transmission dynamics. Moreover, the absence of systematic sero-epidemiological investigations in this region restricts the understanding of pathogen circulation, host exposure patterns, and risk factors associated with transboundary disease spread. The limited availability of baseline data on DHAV, AI, and ND in waterfowl populations along the Indonesia–Malaysia border further constrains the development of effective surveillance systems, vaccination strategies, and One Health-based control measures. Therefore, addressing this gap is essential to support evidence-based decision-making for transboundary animal disease management.

Therefore, this study aimed to determine the seroprevalence and epidemiological patterns of DVH, AI, and ND in waterfowl populations within the Indonesia–Malaysia transboundary region of West Kalimantan, Indonesia. Specifically, the study sought to (i) assess the distribution of antibodies against these major viral pathogens across different districts, species, and age groups; (ii) evaluate clinicopathological findings associated with viral infections; and (iii) generate baseline epidemiological data to support cross-border surveillance and the development of integrated One Health strategies for the prevention and control of transboundary viral diseases in backyard waterfowl production systems.

## MATERIALS AND METHODS

### Ethical approval

This study was reviewed and approved by the Research Ethics Committee of the National Research and Innovation Agency (BRIN), Indonesia, under Animal Ethical Approval No. 071/KE.02/SK/04/2023. All procedures involving animals were conducted in accordance with institutional guidelines for the ethical use and care of animals in research, as well as internationally accepted standards for animal welfare and biosafety.

Prior to field activities, verbal informed consent was obtained from all participating farmers after explaining the objectives of the study, the sampling procedures, and the intended use of the collected data. Participation was entirely voluntary, and confidentiality of farm-related information was maintained throughout the study.

Blood collection was performed aseptically by trained personnel using standard veterinary procedures to minimize pain, stress, and handling time. Approximately 1.5 mL of blood was collected from the wing vein of each bird using sterile syringes and needles. Birds were gently restrained during sampling, and all efforts were made to ensure humane handling and reduce distress. No experimental infection, invasive surgical procedure, or euthanasia was performed as part of this study.

Necropsy and histopathological examinations were conducted only on naturally diseased or deceased birds identified during field investigations. Tissue collection and laboratory procedures were carried out following established biosafety and biosecurity protocols to prevent environmental contamination and pathogen dissemination.

The study complied with national regulations governing animal experimentation and field epidemiological investigations in Indonesia and adhered to One Health principles by integrating animal health, environmental considerations, and public health perspectives during surveillance activities in the transboundary region of West Kalimantan.

### Study period and location

**Figure 1:**
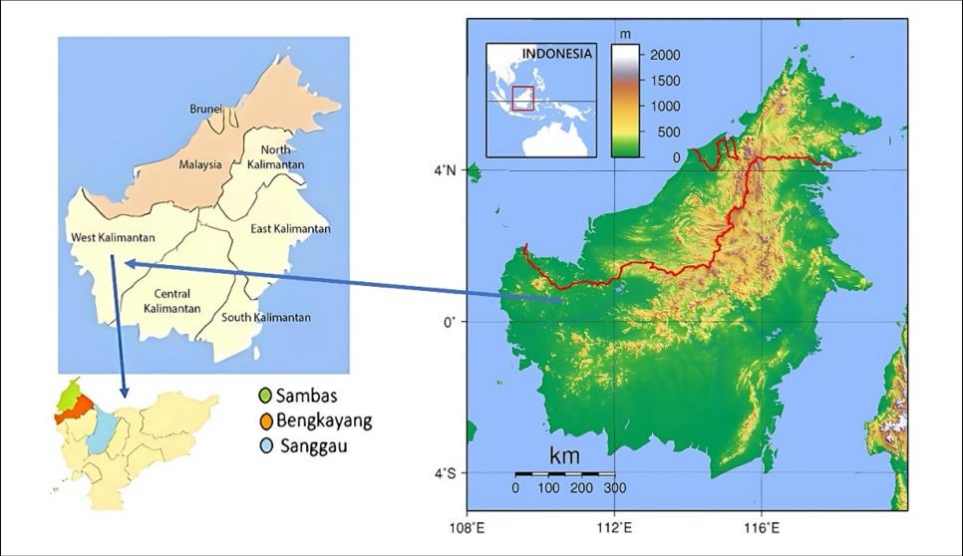
Map of sampling areas showing Sambas, Bengkayang, and Sanggau districts in West Kalimantan Province along the Indonesia–Malaysia border. Source: Original elaboration by the authors; base map from Bing Maps (Open Database License, © Microsoft Corporation).

The study was conducted between 5 and 28 May 2023 in West Kalimantan Province, Indonesia, a transboundary region bordering Sarawak, Malaysia. Sampling was carried out across three districts, namely Sambas, Bengkayang, and Sanggau, selected due to their proximity to the international border and their potential role in cross-border poultry movement and shared ecological interfaces. Figure 1 illustrates the geographical distribution of the sampling locations.

### Study design

A cross-sectional study design was employed using purposive sampling. Flocks were selected based on proximity to border areas, free-range management systems, flock size (>20 birds), and farmers’ willingness to participate, targeting populations at higher risk of exposure to transboundary diseases.

A total of 245 waterfowl blood samples were initially collected from 26 smallholder flocks between 5 and 28 May 2023. Approximately 1.5 mL of blood was collected aseptically from the wing vein of each bird. Of these, 211 serum samples (52 ducks and 159 Muscovy ducks) with sufficient volume for all assays were included in the final analysis. Due to extensive management practices and field constraints, the number of birds sampled per flock varied. Consequently, analyses were conducted at the individual bird level without formal adjustment for intra-flock correlation. The study population consisted of apparently healthy ducks and Muscovy ducks raised under free-range systems. Vaccination history was assessed through structured farmer interviews, and all flocks were reported as unvaccinated at the time of sampling.

### Clinical and pathological examination

Clinical observations and gross pathological examinations were conducted during field sampling to assess the presence of disease in the waterfowl population. During sample collection, all waterfowl were clinically examined for signs of DVH, AI, and ND. Observations focused on identifying typical clinical signs of these diseases, including neurological symptoms, respiratory distress, diarrhea, weakness, and other abnormalities. Birds exhibiting morbidity or mortality underwent further pathological assessment.

Necropsy and histopathological examinations were conducted on a subset of clinically affected birds from selected flocks (notably Farms A and B) where mortality or suggestive lesions were observed. Gross pathological examinations were performed to assess lesions, with particular attention to the liver, air sacs, lungs, intestines, and immune organs such as the bursa of Fabricius.

Tissue samples from affected birds were collected from Farms A and B, located in Sambas and Sanggau, respectively, for histopathological analysis. Samples were fixed in 10% neutral buffered formalin, processed using standard paraffin embedding procedures, and stained with hematoxylin and eosin. Histopathological examination focused on detecting lesions consistent with viral infections, including inflammation, necrosis, hemorrhage, and cellular infiltration in relevant organs.

Cloacal swab samples from Farm A that showed diarrhea were collected for bacteriological examination using standard culture methods and the Analytical Profile Index 20E system (bioMérieux, Marcy-L’Etoile, France) to detect *Salmonella* spp.

### Serological examination

**Enzyme-linked immunosorbent assay for DVH**: A commercial Duck Viral Hepatitis antibody ELISA kit (Catalog No. SL0018Du; SunLong Biotech Co., Ltd., Hangzhou, China) was used to qualitatively assess antibodies against DVH in duck and Muscovy duck serum. The ELISA was performed according to the manufacturer’s instructions.

The microplate wells were pre-coated with DVH-specific antigen to form a solid-phase antigen. Test samples were added to the wells, allowing binding between the antigen and the antibodies present in the samples. Subsequently, horseradish peroxidase-conjugated antigen was added to facilitate antigen–antibody complex formation. After incubation, wells were washed to remove unbound reagents. Tetramethylbenzidine substrate was then added, producing a color change from blue to yellow upon addition of the stop solution. Optical density was measured at a wavelength of 450 nm using a spectrophotometer. Results were interpreted qualitatively based on the kit cutoff value. According to the manufacturer’s documentation, diagnostic sensitivity and specificity were 95% and 98%, respectively.

**Serological testing for AI and ND:** Serological testing was conducted using hemagglutination (HA) and hemagglutination inhibition (HI) methods, following the guidelines of the World Organization for Animal Health [16, 17].

A V-shaped microplate and micro-technique were used to conduct the HA test. The AI antigen (H5N1 subtype clade 2.3.2) and ND antigen (La Sota strain) were obtained from Pusat Veteriner Farma (Pusvetma, Surabaya, Indonesia). Each well was filled with 25 µL of phosphate-buffered saline. An additional 25 µL of antigen was added to the first well and serially diluted twofold across subsequent wells (1/2 to 1/1024). The 12th well served as the red blood cell control. After dilution, phosphate-buffered saline and 1% chicken red blood cells were added, and the mixture was incubated at 26°C (room temperature) for 30 min. The antigen titer was determined, and a 4 HA unit antigen was prepared for the HI assay.

The HI assay was performed using a similar micro-technique. A standard anti-H5N1 antibody (Pusvetma) was used as the positive control. Test samples were serially diluted as in the HA test. In each well, 4 HA units of antigen were added, followed by incubation. Subsequently, 1% chicken red blood cells were added, and the mixture was incubated further. The inhibition of hemagglutination indicated the presence of antibodies against AI or ND, confirming viral exposure [16, 17].

Seropositivity was defined as an HI titer ≥ 1:16 (4 log_2_), in accordance with World Organization for Animal Health recommendations. Geometric mean titers were calculated for seropositive samples where applicable.

### Analysis and management of data

Data were recorded using Microsoft Excel 365 (Microsoft Corporation, Redmond, WA, USA). Herd-level and animal-level prevalence were calculated using the following formula [18]:

Prevalence (%) = (Number of birds that tested positive / Total number of birds examined) × 100

The Chi-square (χ²) test was used to examine differences between species, location, and age groups. The strength of association between potential risk factors and seroprevalence was estimated using odds ratios with 95% confidence intervals [19], calculated using univariate logistic regression with the lowest-risk category as the reference group (Sambas for district, duck for species, and birds aged < 3 months for age).

For variables with sparse data, descriptive comparisons were prioritized to avoid unstable estimates. Statistical significance was set at p < 0.05. Descriptive statistics were used to summarize qualitative and quantitative variables. Due to uneven sampling across flocks, adjustment for intra-flock correlation was not performed, and analyses were conducted at the individual level.

## RESULTS

### Observation of clinical symptoms, gross pathology, and histopathology during sample collection

No clinical signs suggestive of DVH were observed in Farm B. However, clinical signs, gross lesions, and histopathological findings were detected in both Muscovy ducks from Farm A and ducks from Farm B in Sambas and Sanggau districts (Table 1).

**Table 1:** Clinical signs, gross and histopathological lesions, and laboratory findings in waterfowl observed during field sampling in the transboundary area of West Kalimantan, Indonesia.

Farm	Species (Age)	Clinical signs	Gross pathology	Histopathology	Other (laboratory) findings
A	Muscovy ducklings (1 week)	Weakness, lethargy, white diarrhea; 4/10 mortalities	Hemorrhagic intestine	Hemorrhagic pneumonia, lymphoid depletion of the bursa of Fabricius, hemorrhage of the duodenum, ileum, and proventriculitis	*Salmonella* spp. detected, ND seronegative
B	Ducks (3 months)	Emaciation, feather loss, ectoparasites (> 90%), fever, respiratory distress, morbidity (10/100)	Cloudy air sacs, hyperemic lungs and intestines, swollen bursa of Fabricius	Pulmonary, intestinal, and cecal tonsil hemorrhage, focal to diffuse hepatic necrosis, necrosis of cerebrum and cerebellum, hemorrhagic intestine, necrotic bursa of Fabricius	ND seropositive

In Farm A, 10 Muscovy ducks showed weakness, lethargy, and white diarrhea, with four deaths recorded. In contrast, birds in Farm B were emaciated, exhibited feather loss, and showed a high prevalence of ectoparasites (> 90%), accompanied by fever and respiratory distress. Gross examination revealed hemorrhagic intestines in Farm A, whereas Farm B showed cloudy air sacs, hyperemic lungs and intestines, and enlargement of the bursa of Fabricius.

Histological findings in Farm A included hemorrhagic pneumonia, lymphoid depletion of the bursa of Fabricius, and intestinal hemorrhage. In Farm B, lesions included pulmonary and intestinal hemorrhage, hepatic necrosis, cerebral and cerebellar necrosis, and bursal necrosis. These lesions are consistent with systemic viral infections commonly associated with ND or AI rather than DVH.

**Figure 2:**
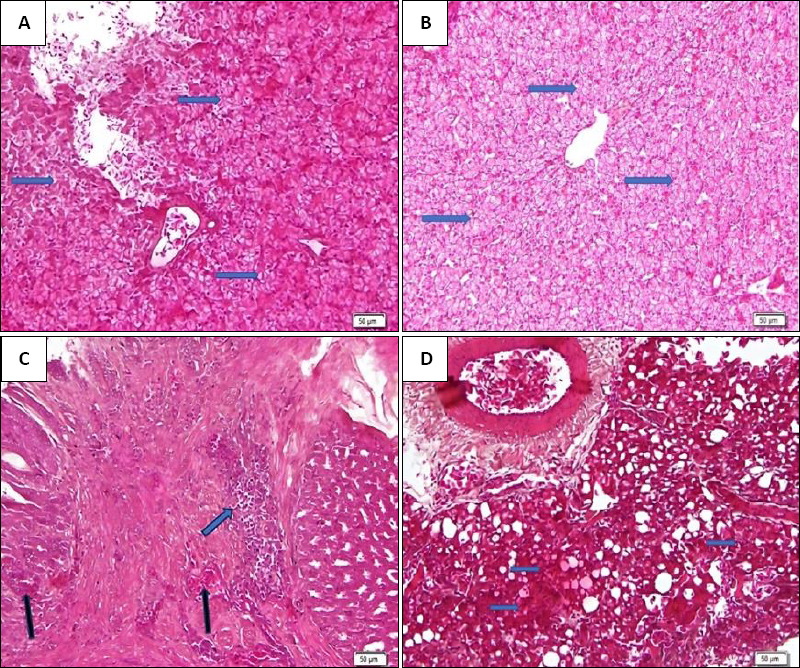
(A) Hemorrhagic liver associated with diffuse hepatonecrosis in Farm A (hematoxylin and eosin stain, 200×). (B) Diffuse hepatic necrosis in Farm A (hematoxylin and eosin stain, 200×). (C) Non-suppurative proventriculitis with focal infiltration of mononuclear cells in the submucosa (Farm B; hematoxylin and eosin stain, 200×). (D) Hemorrhagic interstitial non-suppurative pneumonia (Farm B; hematoxylin and eosin stain, 200×).

Representative lesions are illustrated in Figure 2. The lesion patterns were compatible with viral etiologies, with findings in Farm B suggestive of ND virus infection. In contrast, hemorrhagic changes in Farm A, accompanied by the detection of *Salmonella* spp., may indicate secondary bacterial involvement.

### Seroprevalence of antibodies to DVH, ND, and AI in waterfowl

A total of 211 waterfowl, comprising ducks and Muscovy ducks, were tested for the presence of antibodies against DVH, AI, and ND in the border areas of West Kalimantan, Indonesia.

Table 2 shows that none of the ducks or Muscovy ducks in the three study districts had detectable DVH antibodies at any age. No seropositive cases of DVH antibodies were detected in the sampled waterfowl population (Table 2). All 211 samples tested negative, resulting in an overall seroprevalence of 0.0%. As no positive cases were identified, inferential statistical analysis was not applicable.

**Table 2:** Seroprevalence of DVH antibodies in waterfowl based on district, species, and age group (N = 211).

Variable	Category	Tested (n)	Positive n (%)	Negative n (%)	Prevalence (%)
District	Sambas	93	0 (0.0%)	93 (100%)	0.0
	Bengkayang	62	0 (0.0%)	62 (100%)	0.0
	Sanggau	56	0 (0.0%)	56 (100%)	0.0
Species	Duck	52	0 (0.0%)	52 (100%)	0.0
	Muscovy duck	159	0 (0.0%)	159 (100%)	0.0
Age group	< 3 months	106	0 (0.0%)	106 (100%)	0.0
	3–6 months	60	0 (0.0%)	60 (100%)	0.0
	> 6 months	45	0 (0.0%)	45 (100%)	0.0
Total	—	211	0 (0.0%)	211 (100%)	0.0

No seropositive cases were detected, therefore inferential statistical analysis was not applicable.

Serological evidence of ND exposure was detected in 28 out of 211 samples, yielding an overall seroprevalence of 13.27% (Table 3). Seroprevalence of ND varied slightly across districts, ranging from 11.29% in Bengkayang to 14.29% in Sanggau. Using Sambas as the reference district, no significant differences were observed among districts (p = 0.860). Muscovy ducks showed a slightly lower seroprevalence (12.58%) compared to ducks (15.38%); however, this difference was not statistically significant (p = 0.780).

**Table 3:** Seroprevalence of antibodies to ND in waterfowl in the border areas of West Kalimantan, Indonesia (N = 211).

Variable	Sample size (N)	Negative n (%)	Positive n (%)	OR (95% CI)	p-value
District	—	—	—	—	0.860
Sambas	93	80 (86.02%)	13 (13.98%)	Reference	—
Bengkayang	62	55 (88.71%)	7 (11.29%)	0.78 (0.42–1.43)	—
Sanggau	56	48 (85.71%)	8 (14.29%)	1.03 (0.58–1.83)	—
Species	—	—	—	—	0.780
Duck	52	44 (84.62%)	8 (15.38%)	Reference	—
Muscovy duck	159	139 (87.42%)	20 (12.58%)	1.26 (0.71–2.25)	—
Age (months)	—	—	—	—	0.281
< 3 months	106	95 (89.62%)	11 (10.38%)	Reference	—
3–6 months	60	52 (86.67%)	8 (13.33%)	1.33 (0.78–2.27)	—
> 6 months	45	36 (80.00%)	9 (20.00%)	2.16 (1.29–3.62)	—
Total	211	183 (86.73%)	28 (13.27%)	—	—

Odds ratios were estimated using univariate logistic regression with the lowest-risk category as the reference group. p-values represent overall comparisons using the Chi-square test.

Using birds aged < 3 months as the reference group, those aged 3–6 months (OR = 1.33, 95% CI: 0.78–2.27) and > 6 months (OR = 2.16, 95% CI: 1.29–3.62) showed higher odds of ND seropositivity. Although the overall age effect was not statistically significant (p = 0.281), birds aged > 6 months had numerically higher odds of seropositivity than the reference group.

AI antibodies were detected in 12 of 211 samples, yielding an overall seroprevalence of 5.69% (Table 4). Seropositivity differed significantly across districts (p = 0.014), with the highest proportion observed in Sanggau (12.50%), whereas no positive cases were detected in Bengkayang. Regarding species, Muscovy ducks exhibited a higher AI seroprevalence (6.92%) compared to ducks (1.92%); however, this difference was not statistically significant (p = 0.310).

**Table 4:** Seroprevalence of AI antibodies in waterfowl based on district, species, and age group (N = 211).

Variable	Sample size (N)	Negative n (%)	Positive n (%)	p-value
District	—	—	—	0.014
Sambas	93	88 (94.62%)	5 (5.38%)	—
Bengkayang	62	62 (100%)	0 (0.0%)	—
Sanggau	56	49 (87.50%)	7 (12.50%)	—
Species	—	—	—	0.310
Duck	52	51 (98.08%)	1 (1.92%)	—
Muscovy duck	159	148 (93.08%)	11 (6.92%)	—
Age (months)	—	—	—	0.061
< 3 months	106	96 (90.57%)	10 (9.43%)	—
3–6 months	60	59 (98.33%)	1 (1.67%)	—
> 6 months	45	44 (97.78%)	1 (2.23%)	—
Total	211	199 (94.31%)	12 (5.69%)	—

p-values represent overall comparisons using the Chi-square test. Given the limited number of AI-seropositive cases, odds ratios were not estimated to avoid unstable results.

Age-related differences were not statistically significant (p = 0.061), although descriptively higher seroprevalence was observed in birds aged < 3 months (9.43%) compared with older age groups. Given the relatively small number of AI-seropositive cases (n = 12), inferential comparisons should be interpreted with caution.

## DISCUSSION

### Overview of sero-epidemiological findings

This study investigated the seroprevalence of DVH, AI, and ND in waterfowl (ducks and Muscovy ducks) inhabiting the border region between West Kalimantan, Indonesia, and Malaysia. The sero-epidemiology of infectious diseases in waterfowl, such as DVH, AI, and ND, is critical for understanding disease dynamics and potential threats posed by these pathogens. The findings provide valuable epidemiological insights into the presence and distribution of these economically significant avian diseases in a transboundary context.

### Clinicopathological findings and bacterial involvement

The clinicopathological findings, together with bacteriological examination of cloacal swab samples, indicated the presence of *Salmonella* spp. in Farm A. Histopathological examination of the liver revealed multifocal-to-diffuse hepatic necrosis accompanied by degenerative changes and hemorrhages within the hepatic parenchyma. Similar hepatic lesions have been reported in ducks with *Salmonella* infections, where systemic bacterial dissemination leads to hepatic necrosis and tissue damage [20]. The occurrence of such bacterial infections may also reflect suboptimal farm management and inadequate biosecurity practices that facilitate the introduction and spread of pathogenic bacteria in duck production systems.

### Differential diagnosis and NDV-associated lesions

Non-suppurative proventriculitis and interstitial hemorrhagic pneumonia observed in Farm B were consistent with NDV infection. Viral antigen localization in bronchiolar and alveolar epithelial cells has been demonstrated using immunohistochemical techniques [21]. Highly pathogenic AI is considered an important differential diagnosis of NDV infection due to similarities in clinicopathological findings and tissue tropism. Hemorrhages observed in visceral organs, including the lungs, liver, proventriculus, and intestines, are consistent with previous findings in Peking ducks experimentally infected with highly pathogenic AI in Vietnam [22]. The major differential diagnoses for ND include highly pathogenic AI, infectious bronchitis, infectious laryngotracheitis, and the diphtheritic form of fowl pox. Accurate differentiation among these diseases is essential for establishing a tentative diagnosis [23].

### Absence of DVH antibodies and epidemiological implications

The absence of detectable antibodies to DVH in waterfowl across all age groups and districts in West Kalimantan suggests limited natural exposure to DHAV or the absence of vaccination programs in the study area. Vaccination status was determined through farmer interviews and may be subject to recall bias, which should be considered when interpreting serological findings. In younger birds, maternally derived antibodies may also influence serological interpretation, although their impact under field conditions remains uncertain.

The lack of serological evidence aligns with limited documentation of DHAV circulation in Indonesia, particularly in Borneo. Although DHAV is widely reported in parts of East and Southeast Asia [2], data from the Indonesia–Malaysia border region remain scarce. Regional variations in management practices, biosecurity levels, and poultry movement patterns may contribute to differences in disease occurrence, especially considering the proximity to Sarawak.

Previous studies have demonstrated that DHAV primarily affects ducklings under three weeks of age, often causing acute disease and high mortality rates [24]. The absence of antibodies across age groups highlights the vulnerability of naïve populations, which may face severe outbreaks if the virus is introduced. This finding emphasizes the importance of strengthening biosecurity measures to prevent potential DHAV incursions.

Duck diseases are often multifactorial, and co-infections are common. Liu *et al*. [25] reported co-infection of DHAV with Muscovy duck parvovirus, AI virus, *Escherichia coli*, *Salmonella*, and other pathogens. The absence of DVH detection in this study should be interpreted with caution, as it does not exclude low-level or undetected viral circulation. Detection may also be influenced by sample size, sampling timing, and diagnostic sensitivity [26, 2, 24].

### ND seroprevalence and transmission dynamics

The overall seroprevalence of ND in waterfowl (13.27%, 95% CI: 9.34–18.51) indicates low-level exposure or sporadic circulation of NDV in West Kalimantan. Such circulation suggests that waterfowl may contribute to the epidemiological cycle of the virus, potentially facilitating spillover into domestic poultry [27].

The absence of statistically significant differences in seroprevalence among districts (p > 0.05) indicates relatively uniform exposure risk, possibly due to similar farming practices, bird movement patterns, and environmental conditions. Ducks exhibited a slightly higher seroprevalence (15.38%) compared to Muscovy ducks (12.58%), although this difference was not statistically significant.

In univariate logistic regression, Muscovy ducks had an odds ratio of 1.26 (95% CI: 0.71–2.25), indicating comparable exposure between species. These findings are consistent with reports describing natural NDV infection across multiple waterfowl species. Experimental evidence has demonstrated that Muscovy ducks infected with velogenic NDV (genotype VII) can act as carriers and transmit the virus to in-contact chickens [28].

Age-based analysis showed increasing ND seroprevalence with age, ranging from 10.38% in birds < 3 months to 20.00% in birds > 6 months. Although not statistically significant (p > 0.05), this pattern likely reflects cumulative exposure over time. Similar trends have been reported in endemic regions, where sustained infection pressure results in continuous exposure [29]. Longitudinal studies have also shown persistent circulation of lentogenic avian paramyxovirus-1 strains in waterfowl populations [30]. Furthermore, waterfowl can shed NDV without clinical signs, contributing to silent maintenance of the virus in mixed-species systems [31].

### AI seroprevalence and ecological drivers

The overall AI seroprevalence (5.69%) indicates limited but detectable exposure in the study population. In contrast to ND, significant differences were observed among districts (p < 0.05), with the highest seropositivity in Sanggau (12.50%).

This spatial heterogeneity suggests localized transmission dynamics influenced by ecological factors, poultry trade, and interactions between domestic and wild birds. The higher seroprevalence in Sanggau may be associated with increased interaction between backyard poultry and wetland environments. Similar observations have been reported in Indonesia, where proximity to wetlands facilitates the introduction of viruses via migratory birds [15].

Comparable ecological interfaces have been described in Bangladesh, where wetlands and backyard duck systems support virus circulation at the domestic–wild interface [32]. Although Muscovy ducks exhibited a higher seroprevalence (6.92%) than ducks (1.92%), the difference was not statistically significant and should be interpreted with caution.

Field evidence suggests that Muscovy ducks can be exposed to AI viruses and seroconvert without clinical disease. Studies in Indonesia have reported the detection of non-H5 AI viruses in clinically healthy Muscovy ducks and seropositivity in birds near farms [33, 34]. These findings suggest that Muscovy ducks may contribute to the silent circulation of the AI virus. Waterfowl are recognized as natural reservoirs of influenza A viruses and play a key role in virus maintenance and transmission [35].

### Age-related patterns in AI infection

Age-stratified analysis showed the highest AI seroprevalence in birds aged < 3 months (9.43%), although the difference was not statistically significant (p = 0.061). This trend is consistent with previous studies indicating higher susceptibility and viral shedding in younger birds [14, 15].

The decline in seroprevalence with age may reflect survival bias or prior exposure leading to immunity. Experimental studies have demonstrated greater susceptibility and viral shedding in juvenile ducks, supporting their role as key amplifiers in transmission dynamics [36].

### One Health implications and transboundary risk

From a One Health perspective, the detection of AI antibodies in apparently healthy waterfowl indicates subclinical or past infection events that may pose risks to poultry and human health. Low-pathogenic AI is naturally maintained in waterfowl populations, and viral exchange between wild and domestic birds can lead to the emergence of highly pathogenic strains [37].

The proximity of West Kalimantan to Malaysia increases the risk of cross-border virus introduction, particularly given the active poultry trade and migratory bird pathways. Continued surveillance is essential to safeguard poultry production and mitigate zoonotic risk, as certain AI subtypes, including H5 and H9, have demonstrated zoonotic potential [38].

### Study limitations and future directions

This study has several limitations. First, purposive sampling may introduce selection bias and limit generalizability. Second, clustering at the flock level was not accounted for in the statistical analyses, which may have underestimated the variance. Third, serological testing cannot distinguish between vaccination and natural exposure, and maternally derived antibodies may influence results.

Additionally, cross-reactivity in HI assays may affect antibody interpretation. Sampling was conducted during a single period (May 2023), limiting seasonal inference. The relatively small number of AI-positive cases reduces statistical power and may affect the stability of estimates.

Future studies should incorporate longitudinal designs, larger sample sizes, and molecular approaches to better understand transmission dynamics and the ecological role of waterfowl in maintaining viral pathogens in transboundary regions.

## CONCLUSION

This study provides comprehensive sero-epidemiological evidence on DVH, ND, and AI in waterfowl populations along the Indonesia–Malaysia transboundary region of West Kalimantan. No serological evidence of DVH exposure was detected across all districts, species, and age groups, indicating either the absence of circulating DHAV or limited exposure within the study population. In contrast, antibodies against ND and AI were detected, with overall seroprevalence of 13.27% and 5.69%, respectively, demonstrating ongoing circulation of these viral pathogens in backyard waterfowl systems. Clinicopathological findings further supported viral involvement, particularly lesions consistent with ND and AI, while the detection of *Salmonella* spp. in one flock suggested secondary bacterial infection under field conditions.

The study highlights important epidemiological patterns, including relatively uniform ND exposure across districts and age-related increases in ND seroprevalence, suggesting cumulative exposure over time. In contrast, AI showed spatial variation, with higher seropositivity in specific districts, likely influenced by ecological factors and interactions between domestic and wild birds. The higher, although non-significant, seroprevalence observed in Muscovy ducks indicates their potential role in maintaining subclinical viral circulation. These findings underscore the complex, multifactorial nature of disease dynamics in waterfowl production systems in transboundary environments.

A major strength of this study lies in its integrated multi-pathogen approach, combining serological, clinical, and histopathological assessments to provide a comprehensive understanding of disease occurrence in a high-risk border region. The inclusion of multiple districts and multiple waterfowl species enhances the epidemiological relevance of the findings, while the focus on backyard production systems reflects real-world conditions in which biosecurity is often limited. Additionally, the study contributes baseline data from an underrepresented transboundary region, supporting evidence-based surveillance and control strategies.

Overall, the detection of ND and AI antibodies in apparently healthy waterfowl underscores subclinical infections and the potential for silent viral transmission within and across borders. These findings reinforce the need for strengthened surveillance systems, improved biosecurity practices, and coordinated vaccination strategies targeting ND and AI in backyard production systems. Continued cross-border collaboration and integration of One Health approaches are essential to mitigate transboundary disease risks and safeguard both animal and public health in the region.

## DATA AVAILABILITY

The datasets generated and analyzed during the current study are available from the corresponding author upon reasonable request.

## AUTHORS’ CONTRIBUTIONS

SW, EM, HN, AMA, and DW: Conceived, designed, and coordinated the study. SPP, SW, AMA, AR and DW: Supervised the field sampling, data collection, and laboratory work. EM, SPP, RD, DW, MS, RW, and AA: Data entry, data analysis, and interpretation. EM, SW, HN, AA, and MS: Drafted the manuscript. EM, MS, RD, SW, AR, RW, AA, and HN: Reviewed and edited the manuscript. All authors have read and approved the final version of the manuscript.
